# The X-Linked Inhibitor of Apoptosis Protein Inhibitor Embelin Suppresses Inflammation and Bone Erosion in Collagen Antibody Induced Arthritis Mice

**DOI:** 10.1155/2015/564042

**Published:** 2015-08-04

**Authors:** Anak A. S. S. K. Dharmapatni, Melissa D. Cantley, Victor Marino, Egon Perilli, Tania N. Crotti, Malcolm D. Smith, David R. Haynes

**Affiliations:** ^1^Discipline of Anatomy and Pathology, School of Medical Sciences, University of Adelaide, Adelaide, SA 5005, Australia; ^2^School of Dentistry, University of Adelaide, Adelaide, SA 5005, Australia; ^3^Medical Device Research Institute, School of Computer Science, Engineering and Mathematics, Flinders University, Bedford Park, SA 5042, Australia; ^4^Department of Rheumatology, Flinders Medical Centre and Flinders University, Bedford Park, SA 5042, Australia; ^5^Rheumatology Research Unit, Repatriation Hospital, Daw Park, SA 5041, Australia

## Abstract

*Objective*. To investigate the effect of Embelin, an inhibitor of X-Linked Inhibitor of Apoptosis Protein (XIAP), on inflammation and bone erosion in a collagen antibody induced arthritis (CAIA) in mice. *Methods*. Four groups of mice (*n* = 6 per group) were allocated: CAIA untreated mice, CAIA treated with Prednisolone (10 mg/kg/day), CAIA treated with low dose Embelin (30 mg/kg/day), and CAIA treated with high dose Embelin (50 mg/kg/day). Joint inflammation was evaluated using clinical paw score and histological assessments. Bone erosion was assessed using micro-CT, tartrate resistant acid phosphatase (TRAP) staining, and serum carboxy-terminal collagen crosslinks (CTX-1) ELISA. Immunohistochemistry was used to detect XIAP protein. TUNEL was performed to identify apoptotic cells. *Results*. Low dose, but not high dose Embelin, suppressed inflammation as reflected by lower paw scores (*P* < 0.05) and lower histological scores for inflammation. Low dose Embelin reduced serum CTX-1 (*P* < 0.05) and demonstrated lower histological score and TRAP counting, and slightly higher bone volume as compared to CAIA untreated mice. XIAP expression was not reduced but TUNEL positive cells were more abundant in Embelin treated CAIA mice. *Conclusion*. Low dose Embelin suppressed inflammation and serum CTX-1 in CAIA mice, indicating a potential use for Embelin to treat pathological bone loss.

## 1. Introduction

Rheumatoid arthritis (RA) involves changes in the synovial membrane, including thickening of the synovial lining, persistent infiltration of inflammatory cells, angiogenesis, and production of inflammatory cytokines leading to subsequent cartilage and bone degradation. The joint erosion in RA is associated with increased numbers of bone resorbing osteoclasts [1]. The proliferation of synovial cells and the persistence of inflammatory cells in the RA synovium are associated with a marked reduction in apoptosis [2]. Inducing apoptosis by targeting the upstream pathway with anti-death receptor 5 (DR5) [3] and soluble Fas monoclonal antibody [4] was beneficial in an adjuvant induced arthritis rat model and in a severe combined immunodeficient-HuRAg mouse model, respectively.

Apoptosis is activated through the extrinsic and/or intrinsic pathways. Both pathways converge downstream at the level of executor caspases such as caspases 3, 6, and 7 [5], which are directly responsible for morphological changes in apoptotic cells. The inhibitory apoptotic proteins family (IAPs) regulate both upstream (caspase-9) and downstream caspases (3, 6, and 7) [6] via their baculovirus IAP repeat (BIR) domain. IAP members are also involved in other processes such as protein ubiquitination [7], cell cycle regulation [8, 9], immune function regulation via regulation of cell survival [10], and activation of immune cells. Cellular inhibitors of apoptosis 1 and 2 (cIAP1 and cIAP2) regulate cytokine and chemokine production by macrophages via regulating nucleotide-binding oligomerization domain 2 (NOD2) [11]. Additionally, XIAP regulates innate immunity to* Listeria* infection [12] and is associated with transforming growth factor beta (TGF*β*) [13, 14] and interferes with nuclear factor kappa-light-chain-enhancer of activated B cells (NF*κ*B) and mitogen-activated protein kinases (MAPK) signalling via its BIR1 domain [15].

We have previously reported an increased expression of two IAP family members, XIAP and survivin, in synovial tissues from patients with RA [16] and subsequently demonstrated their modulation by disease modifying antirheumatic drugs (DMARDs) [17], indicating that inhibiting XIAP and/or survivin may be a strategy to treat RA. Supporting this, studies have demonstrated an association between serum survivin and joint erosion [18, 19] and the use of serum survivin levels as an indicator of patient response to infliximab treatment [20]. Suppression of inflammation in an antigen induced arthritis mouse by a Smac mimetic A-4.10099.1 (ABT), an IAP antagonist, supports IAP suppression as a promising target to treat RA [21].

Embelin (2,5-dihydroxy-3-undecyl-1,4-benzoquinone) is a bioactive compound derived from a natural plant,* Embelia ribes*. Its pharmacological actions include antibiotic, antitumour, analgesic, and anti-inflammatory effects [22, 23]. Embelin has been used in acute and chronic models of skin inflammation in mice [24]. It is a cell permeable, nonpeptide small molecule inhibitor of XIAP that can be administered orally; hence it has potential to be an oral therapy for RA [25–27]. Importantly, Embelin can suppress receptor activator of nuclear factor kappa-B ligand (RANKL) induced osteoclastogenesis* in vitro* in RAW 264.7 cells and in myeloma and breast cancer cells [28]; however no studies have assessed the effect of Embelin on bone erosion* in vivo*.

This study investigated the effect of Embelin on inflammation and bone loss in a mouse model of inflammatory arthritis, the collagen antibody induced arthritis (CAIA).

## 2. Methods

### 2.1. CAIA Mice and Treatment Protocol

Experiments were performed in accordance with the Australian Code of Practice for the care and use of animals for scientific purposes (National Health and Medical Research Council) and approved by the University of Adelaide and Institute of medical Sciences Ethics Committees (ethics numbers M-2009-167 and IMVS 75/09, resp.). Induction of arthritis was performed as previously published [29]. Four groups of mice (*n* = 6 per group) consisted of the following: group 1: CAIA without treatment, group 2: CAIA treated with Prednisolone (10 mg/kg/day) [30], group 3: CAIA treated with low dose Embelin (30 mg/kg/day), and group 4: CAIA treated with high dose Embelin (50 mg/kg/day). The number of mice used in each group was kept to a minimum of 6 as the range 6–8 has been suggested for other RA animal models [31]. At day 0, all mice were injected with a 150 *μ*L (1.5 mg total) cocktail of collagen type 2 antibodies (Chondrex kit, Redwood, WA, USA) via the tail vein. On day 3, mice were injected intraperitoneally (i.p.) with 20 *μ*L (10 *μ*g) of lipopolysaccharides (LPS). Embelin in PBS/10% EtOH was administered daily via oral gavage for 7 days (day 4 to day 10). The selected doses administered were in the range reported to give a similar reduction in serum sTNF-*α* to that seen with 30 mg/kg Prednisolone in acute and chronic models of skin inflammation in mice [24]. CAIA mice with no treatment were given the vehicle (PBS/10% EtOH) only. Mice were humanely killed on day 10 and paws were fixed in 10% normal buffer formalin overnight and then were washed with PBS and scanned with microcomputed tomography (micro-CT) before being decalcified and processed for histological evaluation. Serum was collected via cardiac puncture and analysed for CTX-1 using an enzyme-linked immunosorbent assay (CTX-1 ELISA, Ratlaps).

### 2.2. Micro-CT Scans and Image Analysis

To measure bone erosion, micro-CT scans of the paws were performed (SkyScan 1076, Kontich, Belgium)* ex vivo*. The scanning parameters used were as follows: tube voltage 74 kV, tube current 136 *μ*A, isotropic pixel size 17.4 *μ*m, 1.0 mm aluminium filter, and one frame averaging [29].

Cross-sectional images of the front right paws were reconstructed (NRecon, SkyScan) and aligned using the long axis of the paw as reference (Dataviewer, SkyScan). The right radiocarpal joint was chosen for quantitation of the bone volume (BV) [29]. The volume of interest included 40 cross-sections (=0.7 mm) below the epiphyseal growth plate (EGP) to 100 cross-sections (1.7 mm) above the EGP (CTAn V.1.12.04, SkyScan). Grey level images were binarized into bone and nonbone using a uniform threshold (CTAn, SkyScan) [29, 32]. BV (measured in mm^3^) was calculated as the volume occupied by the voxels segmented as bone (CTAn, SkyScan) [29, 32].

### 2.3. Clinical Paw Scoring

Paw scoring was performed daily from day 0 to day 10, by two observers given to each group using a system previously described [33]. In addition, each group was presented randomly for scoring. For each paw a score of 1 was given for each single digit involved; a range of 0–5 was given for swelling of carpal/tarsal and 0–5 for the wrist/ankle [33]. Therefore, the maximal score for each paw could be 15 and the maximal score for each mouse could be 60. Other parameters measured included body weight and daily clinical observations of general health.

### 2.4. Histological Evaluation

#### 2.4.1. Inflammation Score, Cartilage and Bone Degradation Score, and Pannus Score

A score for cellular infiltration (inflammation score), cartilage and bone degradation, and pannus formation was assigned to the hematoxylin-eosin (HE) stained paw specimens according to published methods [29]. Inflammation within the radiocarpal/metatarsal joints was scored as 0 to 3: score 0 ≤ 5% inflammatory cells, score 1 = 6–20%, score 2 = 21–50%, and score 3 = more than 50% inflammatory cells. Cartilage/bone degradation was scored: 0 = normal bone integrity, 1 = mild cartilage destruction, 2 = evidence of both cartilage and bone destruction, 3 = severe cartilage and bone destruction. Pannus formation was scored as 0 = no pannus formation and 1 = pannus formed. Histological scoring was performed by two independent observers that were blinded as to the group allocation.

#### 2.4.2. TRAP Staining

TRAP staining was performed based on a previously published method [34–36]. TRAP positive cells with 3 or more nuclei within the metatarsal and metacarpal joints of the paws were counted using a light microscope with 100x magnification (NIKON D1 digital camera attached to NIKON FXA research microscope, Japan).

#### 2.4.3. XIAP Immunohistochemistry

XIAP expression was detected in paw tissues from all mice using the Vectastain Elite ABC Kit Universal (PK-6200, Vector Labs, Burlingame, CA), according to published methods [37]. The primary antibody was rabbit polyclonal anti-mouse XIAP (Abcam, Ab21278, Sapphire Bioscience Pty. Ltd., NSW, Australia) at 2 *μ*g/mL. Negative controls included omission of primary antibodies or isotype control (rabbit universal isotype IgG, DakoCytomation, Glostrup, Denmark). Sections were stained with 3-amino-9-ethylcarbazole (AEC) dye (Cat. K3469; DakoCytomation, Glostrup, Denmark) and counterstained with hematoxylin and lithium carbonate. Semiquantitative analysis of XIAP immunostaining was performed according to previously published method [38].

#### 2.4.4. Terminal Deoxynucleotidyl Transferase dUTP Nick End Labelling (TUNEL)

TUNEL was performed on paw tissues for apoptosis assessment using an* in situ* cell death detection kit AP (Roche Diagnostic Australia Pty. Ltd., NSW, Australia), as previously published [16]. Tissue was incubated with label solution only for a negative control or with DNA-ase for a positive control for the presence of fragmented DNA. Colour was developed using fast red (Vector Labs, CA, USA) and counterstained with hematoxylin and lithium carbonate.

#### 2.4.5. Serum CTX-1 ELISA

Serum CTX-1 was measured in duplicate for each sample or standard using Ratlaps CTX-1 ELISA according to the manufacturer instructions (Immunodiagnostic Systems, Nordic) [39]. The optical density was measured at 450 nm using a Power-Wave ELISA plate reader and software KC4 (Biotek Instruments, Winooski, VT, USA). Serum CTX-1 concentration was interpolated from the standard curve generated.

#### 2.4.6. Statistical Analysis

Differences in mean values of each parameter between groups were analysed using the Kruskal-Wallis statistical test and differences between two groups were analysed using Mann-Whitney* U* test. Correlation between two parameters was analysed using Kendall's tau b-test. All statistical analysis was performed using SPSS version 20 (Chicago, IL, USA). A *P* value of less than 0.05 was considered statistically significant.

## 3. Results

### 3.1. CAIA Mice Treated with Low Dose Embelin Demonstrated Lower Paw Scores Than Untreated CAIA Mice

**Figure 1:**
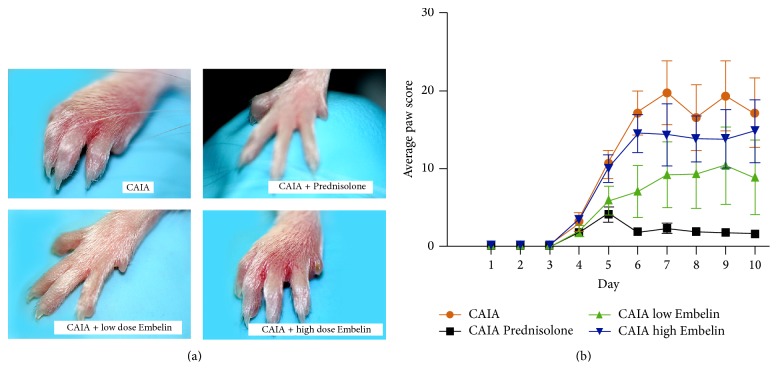
(a) Representative clinical features of mouse paws in CAIA untreated mice, Prednisolone treated CAIA mice, CAIA mice treated with low dose Embelin, and CAIA mice treated with high dose Embelin. (b) Mean clinical paw scores of each study group throughout the experiment. Error bars represent standard error of the mean (SEM).

The front paws in the CAIA mice exhibited inflammation as assessed by clinical paw score (Figure 1(a)). Prednisolone treated CAIA mice (a positive treatment control) consistently demonstrated lower mean paw scores compared to CAIA untreated mice throughout the experiment (*P* < 0.05). CAIA mice treated with a low dose of Embelin also demonstrated markedly lower paw scores throughout the experiment (Figure 1(b)) with statistically significant differences observed on day 6 (*P* < 0.05). Although the mice treated with high dose Embelin demonstrated lower mean paw scores, these were not significant statistically when compared to the CAIA untreated group.

The mice body weights did not differ between groups over the course of the experiment (data not shown).

### 3.2. CAIA Mice Treated with Low Dose Embelin Demonstrated Lower Scores for Inflammation, Cartilage and Bone Degradation, and Pannus Formation

**Figure 2:**
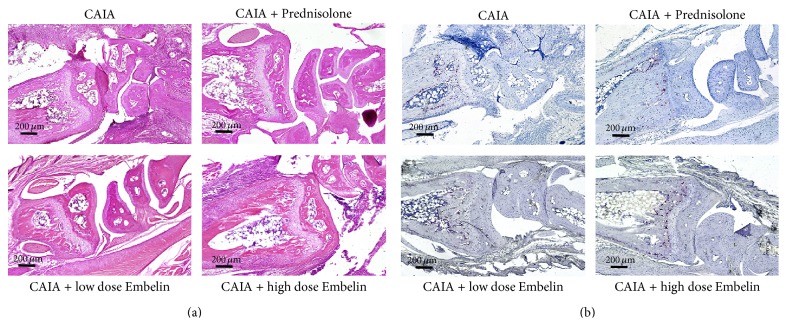
(a) Representative hematoxylin-eosin staining within the radiocarpal joint from each mouse group. (b) Representative TRAP staining (red) in the radiocarpal joint from each mouse group. Hematoxylin counterstaining.

**Figure 3:**
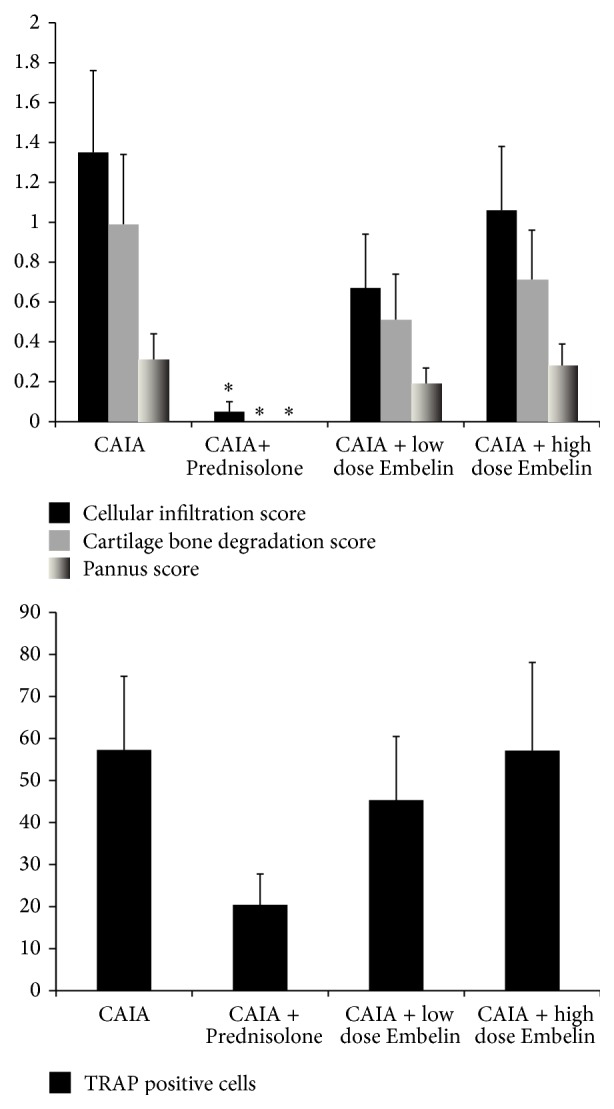
Histological and TRAP scores (obtained using scores as described in Section 2) on paw tissues at day 10. Bars represent mean ± SEM. ^*∗*^ *P* < 0.05.

Histological evaluation of all four paws of all mice showed that CAIA mice treated with low dose Embelin had lower scores for cellular infiltration (*P* = 0.05), cartilage and bone degradation (*P* = 0.071), and pannus formation (*P* = 0.167) compared to CAIA untreated mice. Representative HE stained images of the front right paw from each group are shown (Figure 2(a)). All histological scores were significantly lower in Prednisolone treated CAIA mice compared to untreated CAIA mice (Figure 3).

### 3.3. CAIA Mice Treated with Low Dose Embelin Demonstrated Lower but Not Significantly Different Number of TRAP Positive Osteoclasts

TRAP staining was performed on sections from all four paws of each mouse to identify the number of preosteoclast/osteoclast cells. Representative images of TRAP staining of the front right paw from each group are presented in Figure 2(b). Multiple TRAP positive cells were observed within the joint and pannus region of CAIA mice (mean ± standard error of the mean, 57.27 ± 17.52) and this was reduced with Prednisolone treatment (20.43 ± 7.31) and to a lesser degree in CAIA treated with low dose Embelin (45.33 ± 15.13). Interestingly, CAIA mice treated with high dose Embelin demonstrated similar number of TRAP positive cells to CAIA untreated mice (57.09 ± 21.00) (Figures 2(b) and 3).

### 3.4. There Was No Difference in XIAP Protein Expression between CAIA Mice Treated with Low or High Dose Embelin and CAIA Untreated Mice

**Figure 4:**
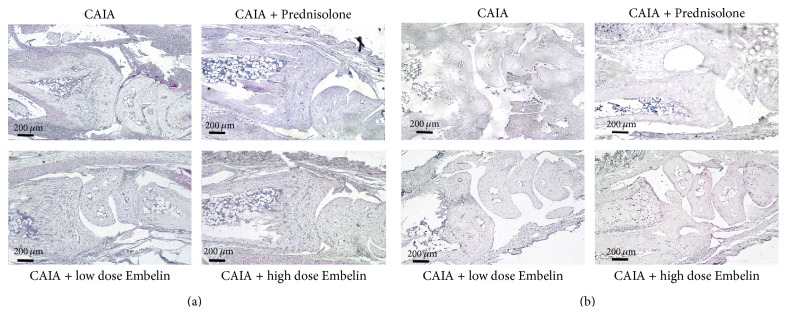
(a) XIAP protein expression (red) in the radiocarpal joint in CAIA mice, in Prednisolone treated mice, in CAIA mice treated with low dose Embelin, and in CAIA mice treated with high dose Embelin. (b) Representative TUNEL staining (indicated in red) in the radiocarpal joint of each mouse group. Hematoxylin counterstaining.

Semiquantitative analysis of XIAP immunostaining revealed that mouse paws from all groups expressed XIAP proteins within cells in pannus, periosteal tissue, articular cartilage, and bone marrow (Figure 4(a)). There was no statistical significant difference in the XIAP expression within the joint space between the groups. Mean (±SEM) score for XIAP was 0.59 ± 0.3 for CAIA untreated mice, 0.85 ± 0.05 for CAIA treated with Prednisolone, 0.62 ± 0.29 for CAIA treated with low dose Embelin, and 0.81 ± 0.39 for CAIA treated with high dose Embelin.

### 3.5. CAIA Mice Treated with Low and High Dose Embelin Demonstrated Increased Numbers of Apoptotic Cells Compared with CAIA Untreated Mice

To investigate if Embelin induced apoptosis in CAIA mice, evidence of fragmented DNA was assessed using TUNEL. Fewer TUNEL positive cells were observed within the pannus of the CAIA mice, compared to prednisolone treated CAIA mice and both low and high dose Embelin treated CAIA mice (Figure 4(b)).

### 3.6. Higher but Not Significant Bone Volume (BV) Was Observed in CAIA Mice Treated with Low and High Dose Embelin Compared to CAIA Untreated Mice

**Figure 5:**
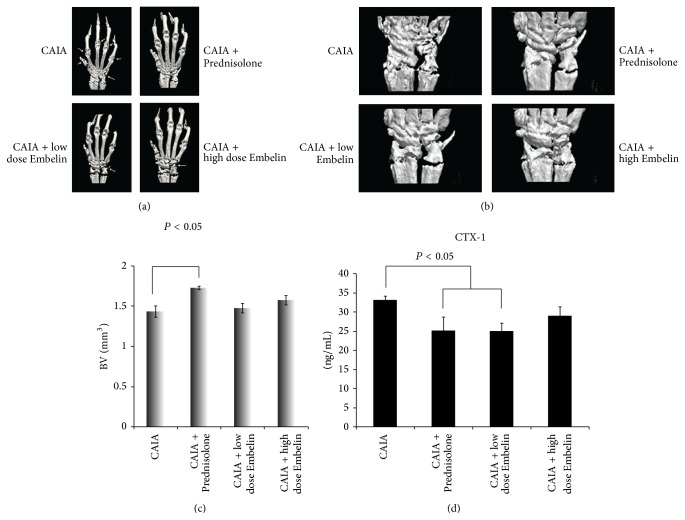
(a) Three-dimensional reconstruction of front right paw in each study group. Arrows point out bone area with erosion. (b) Three-dimensional reconstruction of the radiocarpal region from each mouse group chosen for quantitation of bone volume. (c) Mean bone volume (BV, expressed in mm^3^) analysed by micro-CT in each group. (d) Mean serum CTX-1 level in each mouse group. Error bars represent standard error of the mean (SEM).

Three-dimensional reconstruction of front right paws by micro-CT demonstrated severe osteolysis within their radiocarpal and PIP (proximal interphalangeal) joints of the untreated CAIA mice in marked contrast to Prednisolone treated CAIA mice (Figure 5(a)). There was a significantly higher mean BV within the radiocarpal region in the Prednisolone treated CAIA mice, compared to CAIA untreated mice (1.73 ± 0.02 versus 1.43 ± 0.06, *P* = 0.011) (Figures 5(b) and 5(c)). Although the mean BV was higher in both low and high dose Embelin treated groups (1.47 ± 0.05 and 1.57 ± 0.06, resp.) compared to the CAIA untreated group, there was no statistically significant difference (Figure 5(c)).

### 3.7. CAIA Mice Treated with Low Dose Embelin Demonstrated Significantly Lower Serum CTX-1 Levels Than the Untreated CAIA Mice

Serum CTX-1 was measured as a marker of systemic bone resorption in all groups. Levels were significantly lower in the Prednisolone treated CAIA group (25.28 ± 3.35 ng/mL) as compared to CAIA untreated mice (33.27 ± 0.85, *P* = 0.017). There was significantly lower levels of serum CTX-1 (25.12 ± 1.91) in mice treated with low dose Embelin compared to CAIA untreated mice (*P* = 0.016). Although CTX-1 levels were lower in the high dose Embelin treated group, they were not statistically significant (Figure 5(d)).

### 3.8. Correlation Analysis of Parameters

**Figure 6:**
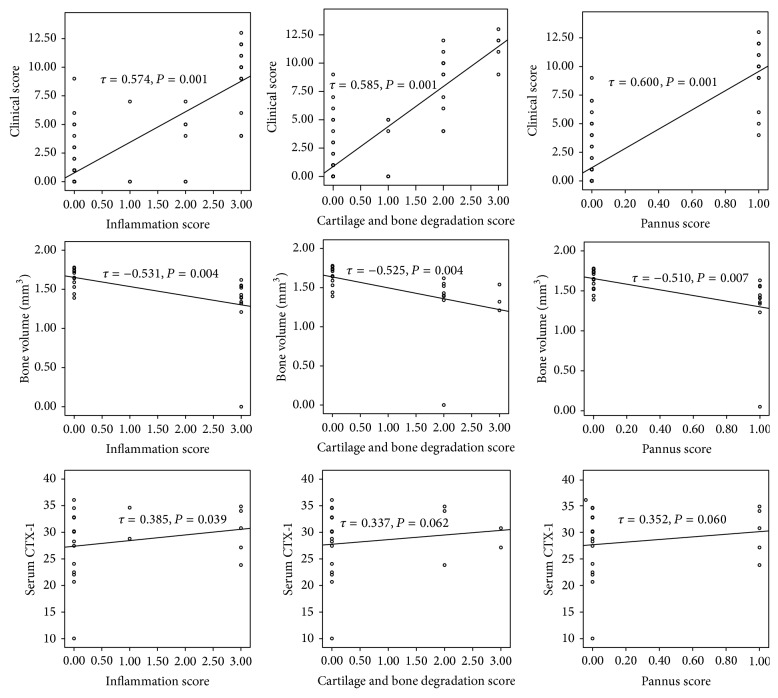
Bivariate correlations between clinical score, bone volume or serum CTX-1 (*y*-axis), and histological scores (inflammation score or cellular infiltration score, cartilage and bone degradation score, and pannus score).

Correlations between clinical and histological scores were made using data from four paws of each of the mice. Only data from the front right paw was correlated with the micro-CT data. Overall, there was a strong and significant correlation between the clinical paw score and each of the histological scores (Figure 6). There was also a significant strong inverse correlation (*τ* = −0.525, *P* = 0.004) between BV as assessed by micro-CT and the cartilage and bone scores assessed histologically. The inverse correlation between BV assessed by micro-CT and the clinical paw score was also strong (*τ* = −0.531) and statistically significant (*P* = 0.004).

## 4. Discussion

This study demonstrates low dose Embelin suppressed joint inflammation and bone erosion in a CAIA model in mice. The findings support a previous study in a model of antigen induced arthritis mice by Mayer et al. [21] where inhibition of IAP using a Smac mimetic suppressed disease activity. In the study by Mayer et al. an antigen induced arthritis was used to assess the initial immune response, whereas the current results demonstrate effects of an XIAP inhibitor on the effector phase of disease. In the antigen induced arthritis model, disease induction involves stimulation of antibody production by the host, via administration of antigen (bovine serum albumin), while in CAIA model this initiation stage is bypassed by the administration of antibodies to collagen antibody type 2. While the IAP inhibitor A-4.10099.1 (ABT) was given intravenously in the Mayer study [21], the XIAP inhibitor (Embelin) used in our study was given via oral gavage, suggesting a potential oral treatment for RA patients. It is important to note that the IAP inhibitor A-4.10099.1 (ABT) was given prior to the disease onset (prophylactic scheme) while in our study the Embelin was assessed for its therapeutic effects with treatment commencing following disease induction.

Mayer et al. [21] elucidated the molecular mechanism involved in suppression of inflammation using a Smac mimetic, IAP antagonist A-4.10099.1 (ABT) with a particular focus on endothelial signaling. However, the study did not report microscopic features of mouse joint damage or assess bone erosion. In the current study, we demonstrate that the XIAP inhibitor, Embelin, used in low dose, suppressed inflammation clinically and microscopically in CAIA mice and we provided evidence of suppression of bone erosion using histological assessment, TRAP counting, micro-CT, and serum CTX-1 assay.

The mechanism by which Embelin suppresses inflammation has been investigated in various* in vivo* models of inflammation. In an LPS induced mouse model of skin inflammation, Embelin was reported to reduce cutaneous TNF-*α* expression [24]. In acetic acid induced colitis Embelin reduced the activity of colonic myeloperoxidase (MPO), lipid peroxides, and serum lactate dehydrogenase as well as significantly increasing the reduced glutathione [40]. In dextran sodium sulphate (DSS) induced colitis in mice, Embelin reduced the mRNA expression of TNF-*α*, IL-1, IL-6, and colonic MPO [41].

The Mayer et al. study suggested that apoptosis was not the mechanism of action of the IAP antagonist A-4.10099.1 (ABT) in abrogating inflammation in antigen induced arthritis mice. They reported that A-4.10099.1 (ABT) regulated the inflammatory processes by attenuation of leucocyte-endothelial cell interaction and decreased TNF-*α* induced activation of TGF*β* activated kinase-1, p38, and cJun N terminal kinase. Smac mimetics can target at least three members of IAPs (cIAP1, cIAP2, and XIAP) and therefore are a broader IAP inhibitor than Embelin. cIAPs have been reported to interfere with the type 2 TNF receptor signaling [6] in addition to directly binding to cleaved caspases 3, 7, and 9 [42].

We demonstrated positive TUNEL staining in the residual inflammatory cells in mice treated with Embelin suggesting that Embelin induced apoptosis in these cells. However, nonresponder mice in the Embelin treated groups demonstrated a very low positivity of TUNEL staining within the pannus suggesting some resistance of inflammatory cells to Embelin induced apoptosis. This correlates with our immunohistochemical evaluation of XIAP protein expression. We observed Embelin reduced XIAP expression within the pannus of responder mice in the group treated with low dose Embelin (*n* = 4), while in the nonresponder mice (*n* = 2) there were very low XIAP levels, low TUNEL staining, and persistence of pannus despite receiving Embelin treatment (data not shown). This suggests that Embelin may only be beneficial if the pannus has high levels of XIAP expression. An* in vitro* study using leukemia cells observed that Embelin reduced XIAP protein expression as detected by western blot [43]. However, a study on glioma cells demonstrated no suppression of XIAP protein despite induction of apoptosis, suggesting suppression of other antiapoptotic molecules such as Bcl2 and Bcl-XL in these cells [44].

The mechanism of action of Embelin in suppressing bone resorption has only been investigated* in vitro*. In this study Embelin suppressed RANKL induced activation of osteoclasts via inhibiting NF*κ*B, inhibition of I*κ*B*α* phosphorylation, and I*κ*B*α* degradation in RAW 267.4 cells [28]. Consistent with this, our study is the first to show Embelin suppression of bone erosion in an* in vivo *model.

Dose-dependent effects of Embelin (10, 30, and 50 mg/kg) have been reported previously in DSS induced colitis in mice [41]; however, in our study the lower dose was more effective than the higher dose. This is also in agreement with a previous study that reported the ED50 of Embelin in LPS induced mouse model of skin inflammation to be 9.8 mg/kg [24]. A similar trend to what we observed with Embelin was seen in a study which investigated the effect of methotrexate in CIA mice [45]. They observed that methotrexate given at 2.5 mg/kg was the most effective dose as compared to 0.1 and 5 mg/kg. The highest dose (5 mg/kg) in that study resulted in a higher clinical score, similar to what we observed with a higher dose of Embelin, which indicates that an optimal dose to reduce the inflammation in arthritis model is not necessarily the highest dose. It is possible that higher dose Embelin failed to suppress inflammation in this model because it has reached a threshold where upregulation of cIAPs occurred as a compensatory mechanism for higher level of XIAP suppression [46]. cIAPs upregulation subsequently can form a complex with TRADD resulting in activation of downstream inflammatory signalling pathways which may maintain inflammation. Further studies to elucidate the involvement of other IAP members in compensatory inhibition of IAP members in this setting are warranted.

In this study, both front paws of each mouse had higher paw scores with most of the mice having highest paw score in the front right paws and hind paws were rarely involved. Based on this observation, micro-CT evaluation was performed on the front right paws. The reduced involvement of hind paws in our CAIA model could possibly be due to the administration of a lower dose of both the cocktail antibody to collagen type 2 and LPS compared to other studies [47]. However, the use of a lower concentration of antibody and LPS resulted in less harmful effects to these mice and possibly is more relevant to the clinical situation in human RA where involvement of all joints is rarely seen.

Our micro-CT analysis showed that mice treated with low dose Embelin exhibited less bone erosion compared to CAIA untreated mice, although the difference was not statistically significant, possibly because a small number (only front right paws from each mouse) was analysed. The evidence of reduced bone damage was supported by the significant decrease in serum CTX-1 level. Nonetheless, BV assessed by micro-CT demonstrated significant positive correlations with both clinical and histological scores and at the same time demonstrated significant negative correlations with serum CTX-1. Overall, this supports the evidence of increased bone damage in untreated CAIA mice compared to CAIA mice treated with Prednisolone and low dose Embelin. Moreover, this confirms micro-CT as an objective quantitative method for assessing bone erosion in a rodent model of arthritis [48, 49].

A recent study involving 108 RA patients receiving low dose Prednisolone and 117 healthy controls has reported that serum CTX-1 (a marker for bone resorption) measured at 0, 3, and 12 months following treatment was significantly lower in the Prednisolone treated group [50]. The current study indicated differences in CTX-1 levels between being untreated and Prednisolone or low dose Embelin treatment, suggesting that serum CTX-1 is sensitive for measurement of bone damage.

Our study supports the induction of apoptosis using low dose Embelin to reduce inflammation and bone erosion in a murine model of inflammatory arthritis. Given the multiple roles of XIAP, further studies on Embelin's mechanism of action to interfere with various signalling pathways in this model are needed. The present study investigated the effect of Embelin in an inflammatory arthritis model that mimics the effector phase of RA; thus studies using Embelin in animal models mimicking the initial stage of RA, such as collagen induced arthritis (CIA) or antigen induced arthritis, will provide further information on the effect of Embelin in the initiation phase of the disease.
